# Pan-genome classification of the virulence spectrum of *Bartonella* and the variation of heme-binding protein virulence factor in isolates from the Tibetan Plateau

**DOI:** 10.1128/spectrum.00465-25

**Published:** 2025-11-11

**Authors:** Jing-peng Liu, Qian Zhou, Chi Zhang, Yi Wu, Xiao-juan Ma, Rui Qi

**Affiliations:** 1 School of Public Health, Lanzhou University, Lanzhou, Gansu, China; 2 Qinghai Center for Disease Control and Prevention 599266, Xining, Qinghai, China; UJF-Grenoble 1, CHU Grenoble, Grenoble, France

**Keywords:** Qinghai-Tibet Plateau, plateau pika, *Bartonella*, pangenome, virulence factors

## Abstract

This study classifies *Bartonella* species based on virulence factors and explores the genomic characteristics of *Bartonella* isolates from the Qinghai-Tibet Plateau to understand their ecological adaptations. PCR detection and isolation culture of *Bartonella* were performed on 50 captured pikas in their habitats on the Qinghai-Tibet Plateau. The Qinghai-Tibet isolates were identified through *gltA* and *rpoB* phylogenetic analysis, ANI analysis, and single-copy homologous gene phylogeny. Reference genomes of *Bartonella* species were analyzed for virulence classification via pan-genome and virulence factor comparisons. *Bartonella* species were categorized into five virulence groups, including a high-virulence group containing *Bartonella henselae, B. quintana, B. kosoyi, B. koehlerae*, and *B. tribocorum. B. bacilliformis* lacked *Trw* and *VirB/VirD4*, while the copy number of *flagella* was significantly higher. *Bartonella grahamii* infected up to 12% of plateau pikas from the Qinghai-Tibet Plateau. *B. grahamii* plateau isolate had the most unique genes (5.6%), with 65.06% of them unknown in function, followed by replication, recombination, and repair (11.64%), and amino acid transport and metabolism (5.48%). The *heme-binding protein* virulence factor in *B. grahamii* plateau isolate exhibited 14 amino acid mutations, leading to changes in the protein-binding sites. *Bartonella* can be classified into five virulence groups. In the high-virulence group, the less common *B. tribocorum* and *B. kosoyi* are included. *Trw* and *VirB/VirD4* gradually replaced ancestral *flagella* genes in *Bartonella* evolution. The isolated strains have more unique genes due to plateau pikas’ unique physiological traits, which may be connected to adaptation to the high-altitude host environment.

**IMPORTANCE:**

*Bartonella* species are emerging zoonotic pathogens capable of infecting a wide range of mammals, including humans. Understanding their virulence and evolution is critical for assessing potential public health risks. This study classifies *Bartonella* species based on virulence factors, revealing species with potential human pathogenicity. The Qinghai-Tibet Plateau, with its extreme environment, may drive bacterial adaptation. By analyzing *Bartonella* isolates from plateau pikas, we identified unique genes and virulence factor variations, providing insights into bacterial evolution in high-altitude hosts. Our findings highlight the significance of pan-genome analysis in identifying pathogenic traits and evaluating zoonotic risks. This study enhances our understanding of *Bartonella* adaptation, aiding in the clinical identification and characterization of highly pathogenic strains.

## INTRODUCTION

*Bartonella* is a pathogen capable of infecting a wide range of mammals, including humans (1), cats and dogs (2), bats (3), and rodents (4), among others. To date, more than 35 *Bartonella* species have been identified (5), with over a dozen confirmed to infect humans and cause disease, including *Bartonella bacilliformis*, *B. quintana*, *B. henselae*, *B. elizabethae*, and *B. grahamii* (6). Among them, *B. bacilliformis*, *B. henselae*, and *B. quintana* have garnered significant attention due to their respective abilities to cause severe Carrion’s disease, cat-scratch disease, and trench fever (7–9). Meanwhile, an increasing number of *Bartonella* species are being found to be pathogenic to humans (such as *B. rattimassiliensis* and *B. alsatica*) (10), and new *Bartonella* species are being discovered as well (11). Therefore, classifying *Bartonella* species based on virulence spectrum is highly relevant to the clinical identification and characterization of highly pathogenic strains. In humans, *Bartonella* infection can lead to a variety of clinical symptoms (1), including intermittent fever and multi-organ inflammation involving the heart, liver, lymph nodes, and other tissues (12), which can even be life-threatening (13). Most human infections with *Bartonella* are zoonotic, typically resulting from animal bites (14) or contact with *Bartonella*-contaminated arthropod feces, or through the bite of blood-feeding arthropods (6). It is confirmed or highly suspected vectors that include sand flies, fleas, ticks, lice, and mites (15). Small mammals serve as the most important hosts for *Bartonella* (16). Reported infection rates range from 24% to 87.5% in North America (17, 18), and endemic foci exist in parts of Africa (7%–60%) and South America (0%–19.2%) with varying reported prevalences (19–24). In Europe, rates have been reported from 18% to 78% (25–27), while reported rates in Asia typically range from 20% to 52% (16, 28–30). In China, the infection rates of *Bartonella* in small mammals also vary by region, with 48% in Yunnan Province (16), 37% in Shanxi Province (31), and 26% in Shaanxi Province (32).

The Qinghai-Tibet Plateau, known as the “Roof of the World,” is the highest and largest plateau on Earth, with an average elevation exceeding 4,000 m and an area of over 2.5 million square kilometers (33). It is characterized by extreme climatic and environmental conditions, including low oxygen levels, high ultraviolet radiation, and cold temperatures (34–36). Over the course of long-term evolution, animals residing on the plateau have developed unique morphological and physiological characteristics (37). The physiological traits of animal hosts can influence the evolution of their resident microbiota and pathogenic bacteria (38). A study found a strain of *Escherichia coli* containing multiple types of virulence factors in the Himalayan marmots on the Qinghai-Tibet Plateau, suggesting that the unique physiological characteristics of plateau animal hosts may induce opportunistic pathogens to develop new virulence traits (39). The plateau pika (*Ochotona curzoniae*) is a small mammal of the family *Ochotonidae* and genus *Ochotona* widely distributed across the Qinghai-Tibet Plateau. It has well adapted to the cold and hypoxic environment of the plateau (40). Fossil evidence suggests that the plateau pika on the Qinghai-Tibet Plateau is very primitive, with all pikas worldwide evolving from this region (41). However, the relationship between *Bartonella* carried by this primitive small mammal and its adaptation to the unique ecological environment of the Qinghai-Tibet Plateau remains unclear.

Pangenome refers to the complete set of genes found within the gene pool of a species (42). Pangenomics provides a new approach to analyzing species divergence and shared gene sets (43). Comparing a newly sequenced bacterial genome with the pangenome, rather than just with the reference genome as in the past, can reveal more insights into the evolutionary and variation characteristics of the bacterium (44). Through pan-genome and virulence factor comparative analysis, this study classified *Bartonella* into distinct groups and revealed changes in virulence factors during its evolution. Additionally, this study investigated the infection status of *Bartonella* in plateau pikas and conducted whole-genome sequencing (WGS) on the isolated *Bartonella* strain, exploring the unique genes, evolutionary characteristics, and virulence factor profiles of *Bartonella* in plateau pikas from the Qinghai-Tibet Plateau. This study aims to identify *Bartonella* species with potential pathogenicity to humans through virulence factor analysis while also exploring the possible impact of the unique environment of the Qinghai-Tibet Plateau on *Bartonella* virulence factors.

## RESULTS

### Pangenome analysis of the *Bartonella* and comparative analysis of virulence factors

**Fig 1:**
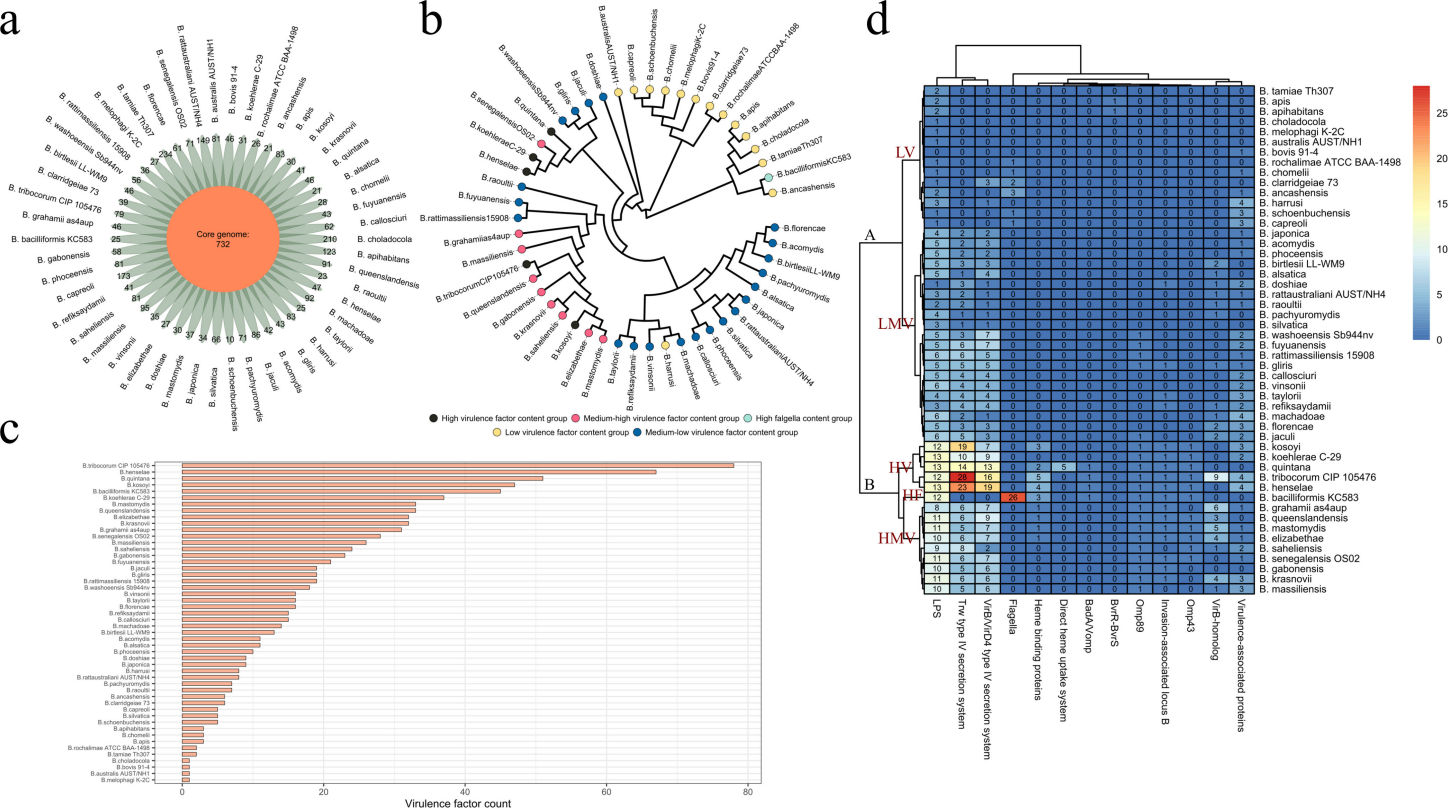
Pangenome and virulence factor analysis of *Bartonella*. (**a**) Core genome and unique genome of 50 *Bartonella* species. (**b**) Phylogenetic tree based on shared homologous single-copy genes of *Bartonella*, with differently colored points representing different virulence factor groups. (**c**) Bar chart showing the virulence factor content in different *Bartonella* species. (**d**) Identification of virulence factors in different *Bartonella* species. Based on their virulence factor profiles, the 50 *Bartonella* strains were clustered into 5 groups: HV factor content group, HMV factor content group, HF group, LMV factor content group, and LV factor content group.

A pan-genome analysis of 50 different *Bartonella* species genomes revealed a pan-genome size of 5,794 genes, with a core genome size of 732 genes (Fig. 1a). The phylogenetic tree of the homologous single-copy genes from these 50 *Bartonella* strains showed that species capable of causing human infections, such as *B. quintana*, *B. henselae*, and *B. koehlerae*, clustered within the same branch. In contrast, *B. bacilliformis*, another species that causes human infections, was positioned on a more ancient branch of the phylogenetic tree (Fig. 1b). There was a significant difference in the number of virulence factors among different *Bartonella* species. The five *Bartonella* species with the highest number of virulence factors were *B. tribocorum* (78), *B. henselae* (67), *B. quintana* (51), *B. kosoyi* (47), and *B. bacilliformis* (45) (Fig. 1c). Different *Bartonella* species carried varying numbers of different types of virulence factors. The 50 *Bartonella* species were grouped into two major clusters, Cluster A and Cluster B, based on the detection of different virulence factors. Cluster A included *B. tamiae*, *B. apis*, *B. apihabitans*, and other species with lower virulence factor content, while Cluster B included *B. kosoyi*, *B. quintana*, *B. henselae*, *B. bacilliformis*, and *B. grahamii*, which had higher virulence factor content. In Cluster B, except for *B. bacilliformis*, the other species had higher copy numbers of lipopolysaccharide (*LPS)*, *Trw*, and *VirB/VirD4*. Cluster A could be further divided into a low virulence (LV) factor group (LV) and a medium-low virulence (MLV) factor group. Cluster B could be divided into a high virulence (HV) factor group, a medium-high virulence (MHV) factor group, and a high flagella (HF) group. The HV group includes five species: *B. kosoyi*, *B. koehlerae*, *B. quintana*, *B. tribocorum*, and *B. henselae*. The HF group included only *B. bacilliformis*, which, compared to other species in Cluster B, exhibited unique virulence factor characteristics. Notably, *B. bacilliformis* lacked Trw and VirB/VirD4, while having a significantly higher copy number of *Flagella* than the other strains (Fig. 1d).

### *Bartonella* infection in plateau pikas of the Qinghai-Tibet Plateau

**Fig 2:**
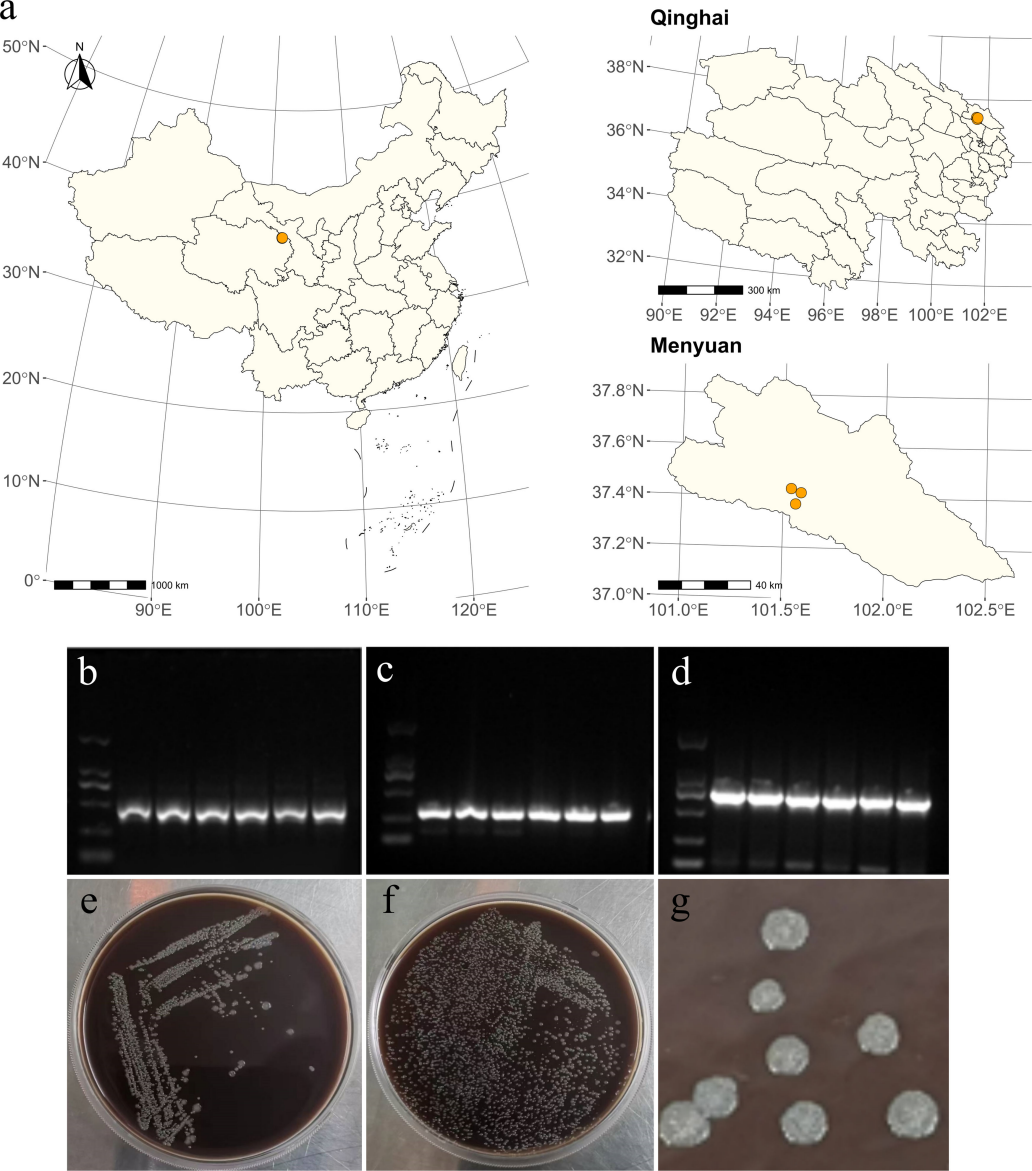
Sampling sites of Plateau pikas and isolation and culture of *Bartonella*. (**a**) The plateau pikas in this study were captured from Menyuan County, Qinghai Province, located in the northeastern part of the Qinghai-Tibet Plateau. (**b**) Results of the PCR product (455 bp *ITS* fragment) analysis on 1. 2% agarose gel; (**c**) 379 bp *gltA* fragment; (**d**) 852 bp *rpoB* fragment; (**e**) the growth and colony status of *Bartonella* species in TSA medium containing 5% defibrinated sheep blood by smearing culture; (**f**) culture of plate streaking; and (**g**) morphology of the colony.

In this study, the pikas were captured from Menyuan County in Qinghai Province, China. In this region, the pikas live around human villages, having close contact with humans. The specific location is shown in Fig. 2a. Among the 50 pika samples, 6 (12.0%) tested positive for *Bartonella* (Fig. 2b through d). *Bartonella* isolation and culture were performed on the PCR-positive samples, and the resulting bacterial colonies exhibited the distinctive morphological characteristics of *Bartonella*: small, round, raised, grayish-white, and slightly translucent (Fig. 2e through g). To further determine the species classification of the isolated strains, the *rpoB* and *gltA* genes of the isolates were PCR-amplified and sequenced. Sequence alignment results of both the *rpoB* and *gltA* genes indicated that the isolates belonged to the species *B. grahamii* (Tables S2 and S3).

**Fig 3:**
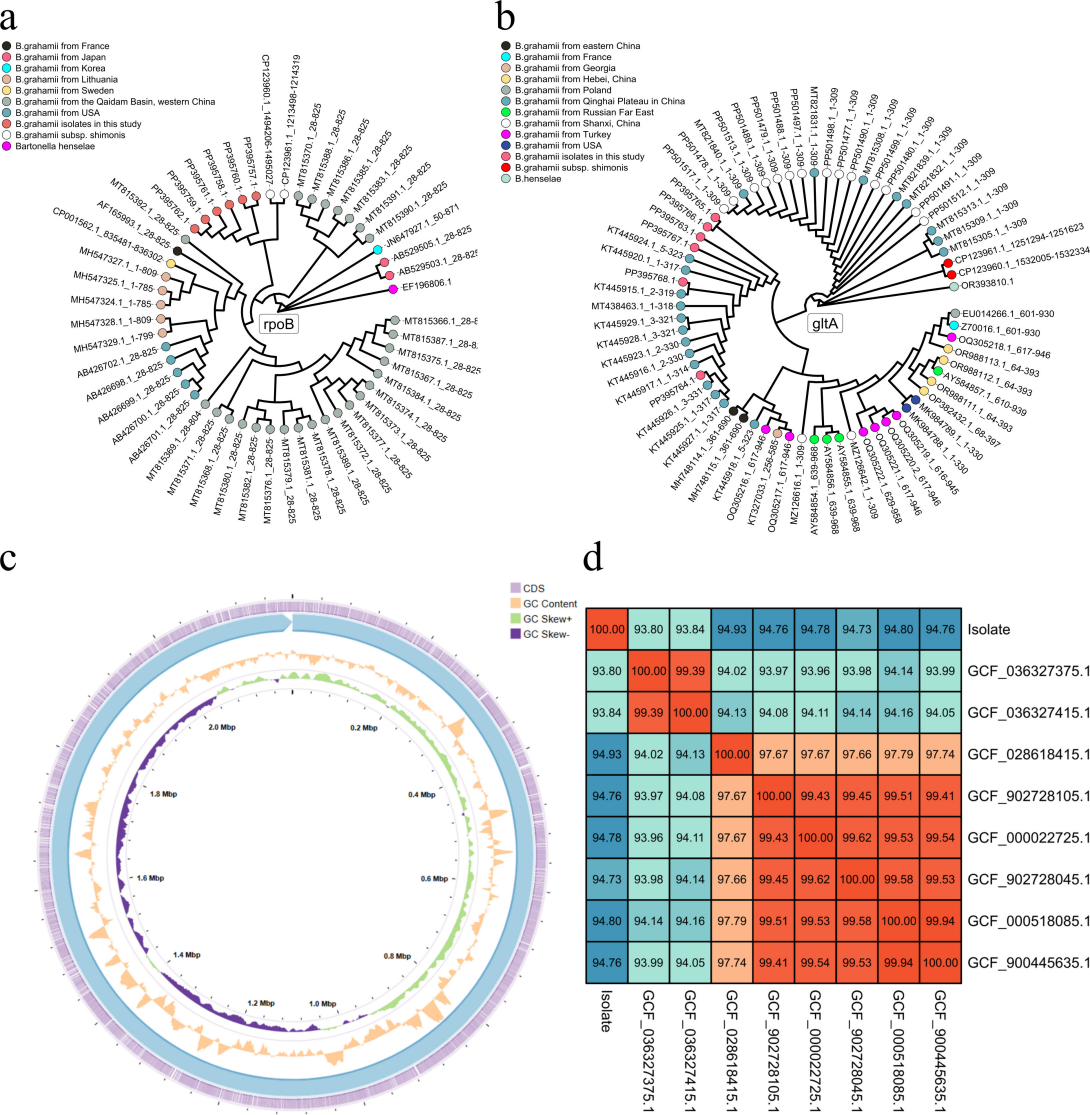
Evolutionary characteristics, general genomic features, and average nucleotide identity (ANI) of *Bartonella* isolates. (**a**) A phylogenetic tree was constructed based on the *rpoB* gene sequence of the isolates, with different colors representing samples from different regions. (**b**) A phylogenetic tree was constructed based on the *gltA* gene sequence of the isolates. (**c**) Genomic circular map of the isolates, with GC skew, GC content, genome, and coding regions represented from the inner to outer rings. (**d**) ANI between the isolates and *B. grahamii* from the NCBI database.

By constructing phylogenetic trees for the *gltA* and *rpoB* genes, some differences between the two results were observed. In the phylogenetic tree based on the *rpoB* gene, the isolates from this study clustered closely with *B. grahamii* subsp. *shimonis* (Fig. 3a). However, in the phylogenetic tree based on the *gltA* gene, the isolates showed a more distant evolutionary relationship with *B. grahamii* subsp. *shimonis* (Fig. 3b). To further determine the evolutionary characteristics and virulence factors of the *Bartonella grahamii* isolates from plateau pikas, one isolate was selected for WGS.

### General genomic characteristics of the *B.a grahamii* isolate from the Qinghai-Tibet Plateau

After assembling the sequencing data, one chromosome and one plasmid were obtained. The chromosome sequence was 2,197,397 bp in length with a GC content of 38.03%. Annotation using Prokka identified 1,912 coding sequences (CDSs; Table S4). The GC content, GC-skew, and CDS positions across different regions of the genome are shown in Fig. 3c. The plasmid sequence was 31,902 bp in length with a GC content of 36.49%. The average nucleotide identity (ANI) analysis of the isolated strain and 8 *B. grahamii* strains from the NCBI database revealed that the 9 *B. grahamii* strains could be divided into 3 groups. The isolated strain formed group A alone; two *Bartonella grahamii* subsp. *Shimonis* strains formed group B, and the remaining six strains formed group C. The ANI between the isolated strain and the eight *B. grahamii* strains was less than 95%. The ANI values with the two *Bartonella grahamii* subsp. *Shimonis* strains were 93.80% and 93.84%, respectively, which was lower than the ANI with the six *B. grahamii* strains in group C. Within group B, the ANI between the two *Bartonella grahamii* subsp. *Shimonis* strains was close to 100%, while their ANI with other strains was less than 95%. Within group C, the ANI among the *B. grahamii* strains was greater than 97%, and among the five strains (excluding GCF_028618415.1), the ANI was greater than 99% (Fig. 3d).

### Pangenome analysis of *B. grahamii* and genomic characteristics of the isolate from the Qinghai-Tibet Plateau

**Fig 4:**
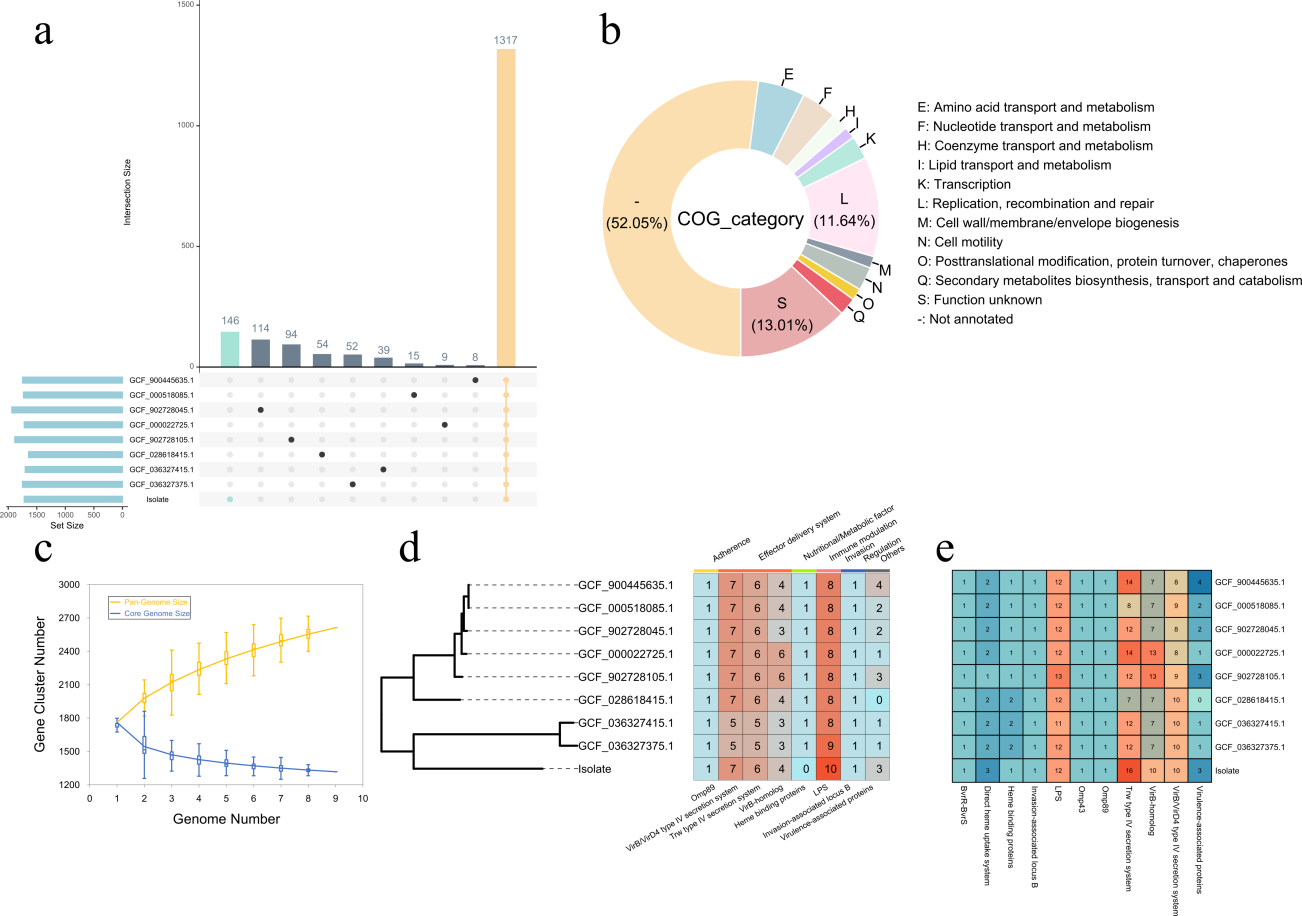
Pangenome and virulence factor analysis of *B. grahamii*. (**a**) Pangenome content of *B. grahamii* and the number of unique genes in different strains. (**b**) Clusters of Orthologous Gene functional classification of unique genes in the isolates. (**c**) Pangenome and unique genome rarefaction curves of *B. grahamii*, with the yellow and blue curves representing the increase in pangenome size and the decrease in core genome size as more of the full genome of *B. grahamii* is added. (**d**) Phylogenetic tree of homologous single-copy genes and the identification of virulence factors in different strains of *B. grahamii* at a 90% identity threshold. (**e**) Virulence factors content in different strains of *B. grahamii* at an 80% identity threshold.

The pan-genome analysis of the 9 *B. grahamii* strains revealed a total of 2,596 gene families, with 1,317 genes constituting the core genome shared by all 9 strains. Regarding the unique genomes of each *B. grahamii* strain, the isolated strain had the highest number of unique genes, with 146 genes (5.6%, 146/2,596) present only in the isolated strain (Fig. 4a). Among the unique genes of the isolated strain, 65.06% were of unknown function, followed by genes associated with replication, recombination, and repair (11.64%), amino acid transport and metabolism (5.48%), and nucleotide transport and metabolism (4.11%) (Fig. 4b). The exponential growth and exponential decline models constructed for the pangenome and core genome indicated that for *B. grahamii*, the pangenome continued to increase as the number of genomes increased (Fig. 4c).

The phylogenetic tree constructed using the shared single-copy homologous genes of *B. grahamii* genomes revealed that the *B. grahamii* strains clustered into two main clades. This result was similar to the phylogenetic tree constructed with the *rpoB* gene and the ANI analysis. The isolate clustered separately with the two strains of *Bartonella grahamii* subsp. *shimonis* (Fig. 4d). The virulence factor analysis revealed that *B. grahamii* contained a total of seven categories of virulence factors: adhesion, effector secretion systems, metabolic systems, immune modulation, invasion, regulation, and others. Effector secretion systems represent the predominant virulence factor type in *B. grahamii*, including three specific virulence factors: *VirB/VirD4*, *Trw*, and *VirB-homolog*. Additionally, *B. grahamii* harbors a relatively high number of immune modulation-related virulence factors, primarily *LPS*. In contrast, virulence factors associated with adherence, nutritional/metabolic functions, and regulation are less prevalent in *B. grahamii*. Among these, the type IV secretion system and *LPS* were found to be abundant across all *B. grahamii* strains. The virulence factors *Omp89* and *Invasion-associated locus B* had identical occurrence frequencies in all nine strains, while other virulence factors exhibited some variation between different strains (Fig. 4d and e). Although the isolated strain clusters with *Bartonella grahamii* subsp. *Shimonis* in the phylogenetic tree, the isolated strain contained a higher number of virulence factors, including *VirB/VirD4*, *Trw*, and *Virulence-associated proteins*, compared to the two *Bartonella grahamii* subsp. *Shimonis* strains (Fig. 4d).

**Fig 5:**
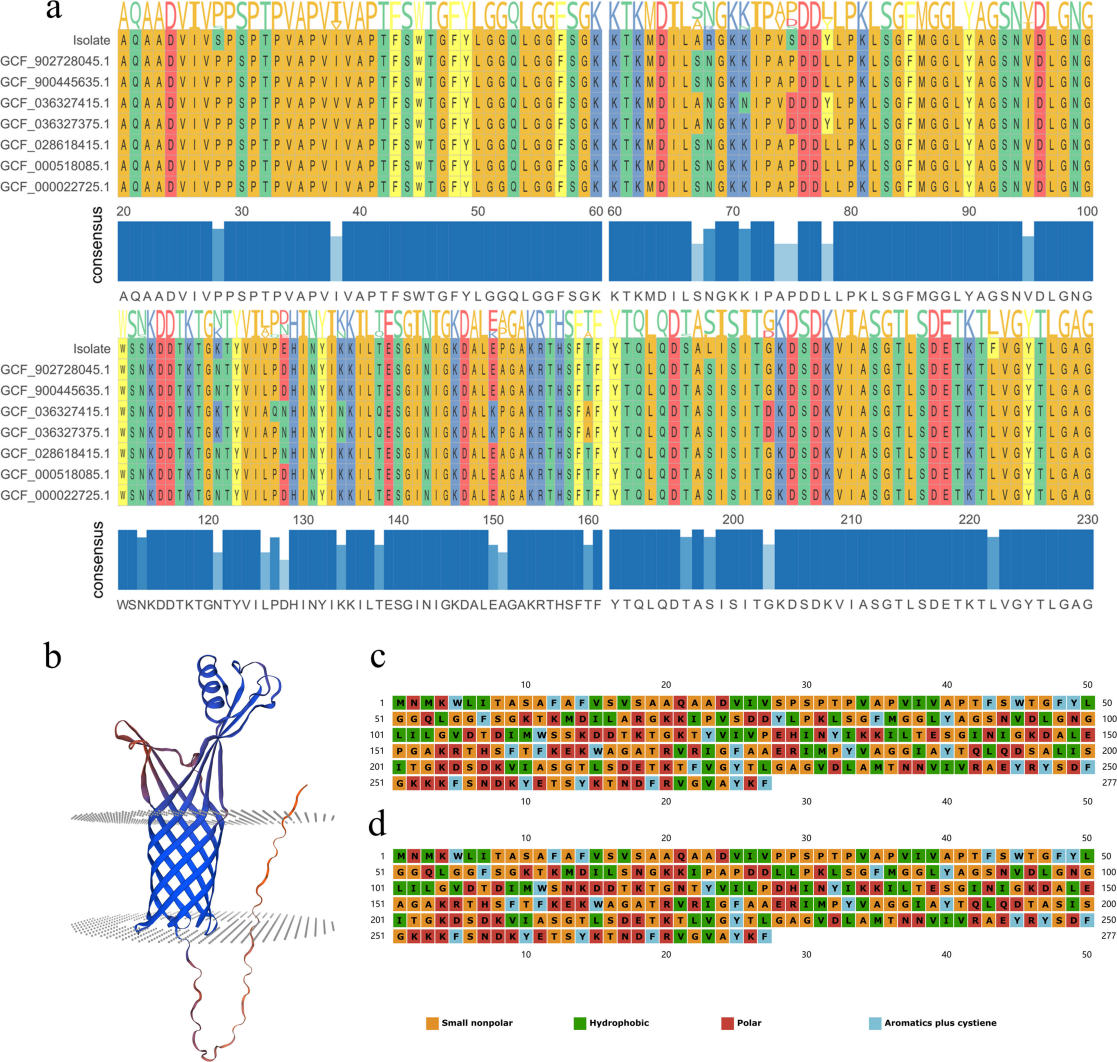
Analysis of *Hbp* mutation sites in *Bartonella* isolates. (**a**) Comparison of amino acid sequences of the *Hbp* virulence factor in *Bartonella* isolates and other strains. The blue bars below indicate the degree of amino acid identity at different sites of the *Hbp* across different strains, with the specific sequence comparison shown above. (**b**) Predicted tertiary structure model of the Hbp in *Bartonella* isolates, with the gray plane representing the transmembrane region. (**c**) Types of amino acids constituting the mutated *Hbp* in *Bartonella* isolates. (**d**) Types of amino acids constituting the *Hbp* before mutation.

Amino acid sequence alignment revealed 14 amino acid mutation sites in the *heme-binding protein* (*Hbp*) virulence factor of the isolated strain (Fig. 5a; Table S5). For instance, at position 28, proline mutated to serine; at position 68, asparagine mutated to arginine; at position 196, threonine mutated to serine; and at position 198, serine mutated to leucine (Fig. 5a; Table S5). Among these, five amino acid mutations resulted in changes in the type of amino acids. For example, at position 74, a non-polar amino acid mutated to a hydrophobic amino acid, and at position 113, a polar amino acid mutated to a non-polar amino acid (Fig. 5c and d; Table S5). Protein tertiary structure prediction of the *Hbp* of the isolated strain revealed the presence of a transmembrane region (Fig. 5b). Comparing the ligand-binding sites of the mutated *Hbp* with the original *Hbp*, it was found that the mutated *Hbp* had one less ligand-binding site. Additionally, the volume and depth of the protein’s active pocket were reduced (Table S6).

## DISCUSSION

In this study, the virulence factor profiles of 50 species of *Bartonella* were mapped, dividing them into groups based on their virulence factor content: high virulence factor group, medium-high virulence factor group, medium-low virulence factor group, low virulence factor group, and a high-flagellum group that only includes *B. bacilliformis*. Among the major *Bartonella* species causing human disease, *B. bacilliformis* (causing Carrion’s disease), *B. henselae* (causing cat scratch disease), and *B. quintana* (causing trench fever) possess well-defined clinical significance and are of major importance. Notably, *B. bacilliformis* has been classified into a separate group due to its highly flagellated properties. Furthermore, within the group exhibiting high virulence factors, in addition to the primarily pathogenic *B. henselae* and *B. quintana*, *B. koehlerae*, *B. kosoyi*, and *B. tribocorum* are also included. *B. koehlerae* exhibits a high prevalence of infection in cats and can cause infective endocarditis in humans, with clinical manifestations similar to diseases caused by *B. henselae* and *B. quintana*, although it is relatively uncommon in humans (45). For *B. tribocorum*, reports of PCR-confirmed infections in humans are scarce; however, evidence suggests its potential pathogenicity, such as one study that successfully isolated *B. tribocorum* from an acutely febrile patient (46). In contrast, *B. kosoyi*, a novel species identified in 2020 (11), has not yet been reported to infect humans to date. Therefore, based on virulence factor profiling analysis, this study demonstrates that *B. bacilliformis* exhibits distinct characteristics regarding flagella-associated factors. *B. henselae* and *B. quintana* share a similar virulence factor profile with other species in the high-virulence group (such as *B. koehlerae* and *B. tribocorum*), suggesting potential common molecular bases for their pathogenicity. However, we note observed differences in the actual frequency and severity of disease manifestation among the species (e.g., severe human cases are primarily caused by *B. bacilliformis*, *B. henselae*, and *B. quintana*). This indicates that actual pathogenicity is the result of complex regulation by multifaceted factors, including host factors, transmission routes, and ecological contexts. Consequently, integrating virulence trait analysis with population-level clinico-epidemiological evidence is crucial for comprehensively assessing the pathogenic potential of species. This integrated approach also represents a key direction for future research aimed at elucidating pathogenic mechanisms and ecological adaptations.

In the phylogenetic tree constructed from homologous single-copy genes of *Bartonella*, the five species of the high virulence factor group, including the human-infecting species *B. henselae*, *B. quintana*, and *B. koehlerae*, cluster together. *B. bacilliformis*, a more ancient species in the evolutionary tree, has a unique virulence factor profile, with a significantly higher number of *flagellum* virulence factors than other *Bartonella* species. On the other hand, the *Trw* and *VirB/VirD4* virulence factors, which are commonly found in other *Bartonella* species, are absent in *B. bacilliformis*. Both *Trw* and *VirB/VirD4* are components of the Type IV Secretion System (T4SS), which plays a critical role in numerous pathogens. The T4SS mediates the internalization into erythrocytes and inhibition of host cell apoptosis via the injection of *Bep* effector proteins (e.g., *BepA*), thereby facilitating the establishment of persistent bacteremia (47). *Vomp*-mediated collagen adhesion creates a localized microenvironment for the T4SS, synergistically promoting bacterial invasion into the endothelium. This process is essential for valvular colonization in endocarditis and vascular proliferation in bacillary angiomatosis (48). The latter is directly associated with the bacterial activation of the VEGF signaling pathway (49): surface proteins such as *Vomp/BadA* induce the overexpression of host vascular endothelial growth factor (VEGF), driving pathological neovascularization. This dynamic virulence network—encompassing *Vomp* phase variation, T4SS effectors, and angiogenic signaling—collectively sustains bacterial immune evasion within the host. Ultimately, this results in the characteristic triad of manifestations: recurrent fever, multi-organ vasculopathy, and endocardial damage (50). Previous studies have shown that the loss of *flagellum* and the appearance of *Trw* in *Bartonella* may occur simultaneously. *Trw* plays a role in promoting erythrocyte invasion and may functionally replace the *flagellum* (51). Considering the ancient position of *B. bacilliformis* in the phylogenetic tree, this study suggests that other *Bartonella* species evolved *Trw* and *VirB/VirD4* during their evolution from *B. bacilliformis*, replacing some of the functions of the original *flagellum*. Additionally, the facultative intracellular parasitism characteristic of *Bartonella* likely limited the mobility of the *flagellum*, leading to the gradual loss of *flagellum* virulence factors during evolution and the emergence of new *Bartonella* species that do not contain *flagella*.

This study found that the infection rate of *Bartonella* spp. in plateau pikas within the surveyed area was 12%, which aligns with findings reported by Xiang-Rao Hua et al. in their investigation of *Bartonella* infections among plateau pikas in Qinghai Province (52). Menyuan County, located in the northeastern part of the Tibetan Plateau, has a typical plateau continental climate. As the most abundant small mammal in the Tibetan Plateau region, the plateau pika has close interactions with local residents (53). Therefore, the infection of pathogenic microorganisms in plateau pikas not only affects the health of other animals within the same ecosystem but also has a direct impact on the health of residents in surrounding areas. In this study, *Bartonella*-positive samples were isolated and cultured, and further species identification revealed that the infected species was *Bartonella grahamii. B. grahamii* was first isolated from the blood of a small woodland mammal in the UK and had shown zoonotic potential (54). *B. grahamii* has been isolated from patients with neuroretinitis, bilateral retinal branch artery occlusion, and chronic lymphocytic leukemia (55). In this study, *B. grahamii* was classified into the high-medium virulence group. Human residential areas in the Menyuan region overlap with the habitat of the plateau pika. While this study detected *B. grahamii* in plateau pikas, direct evidence of zoonotic transmission to humans in this region remains undocumented. Given the species’ known pathogenicity in humans globally and the observed human-pika habitat overlap, future surveillance should prioritize investigating potential spillover risks through seroprevalence studies or pathogen genomics in local populations.

The phylogenetic tree constructed using the *rpoB* gene and the homologous single-copy gene tree both show that the isolated strain is relatively closely related to *B. grahamii* subsp. *shimonis* from Czech *Glis glis* (56). However, the ANI analysis indicates that the ANI between the isolated strain and other strains is below 95%, suggesting that the isolated strain cannot be classified as *B. grahamii* subsp. *shimonis*. It is likely to represent a distinct variant of *B. grahamii* specific to the Tibetan Plateau. The pan-genome is the complete set of genes present within a species, composed of the core genes shared by all individuals, accessory genes present in some individuals, and unique genes found only in single individuals (57). In this study, pan-genome analysis revealed that the isolated strain possesses the richest set of unique genes, most of which are of unknown function. Previous studies have shown that unique genes often provide bacteria with selective advantages, including ecological niche adaptation, antibiotic resistance, and the ability to colonize new hosts (58). Considering that the isolated strain comes from a high-altitude mammalian host, which has physiological traits different from those of forest or grassland mammals, it is hypothesized that these unique genes may be related to adapting to the physiological characteristics of high-altitude animal hosts.

The distribution of virulence factors in *B. grahamii* shows relatively minor differences. Virulence factors such as *Omp89*, *VirB/VirD4*, *Trw*, and *IalB* are similarly detected across all *B. grahamii* strains. The virulence factor *Hbp* in the isolate exhibited multiple mutation sites. These mutations lead to a reduction in the number of binding sites for ligands, as well as a decrease in the size and depth of these binding pockets. Proteins execute their biological functions through interactions with other molecules, typically occurring at binding sites located in the recessed regions on the surface of the protein, known as pockets (59). The changes in the *Hbp* binding sites in the isolate may be related to the unique physiological characteristics of its high-altitude host. Pathogens require iron during growth and colonization and have evolved a variety of mechanisms to acquire iron from their host, with many pathogens primarily obtaining iron from the host’s heme (60). Heme is the largest iron storage reservoir in the host and a major source of iron for many bacterial pathogens (60). In most pathogens, highly affinity-optimized Hbps have evolved to acquire heme (61). Studies have shown that in hypoxic high-altitude environments, high-altitude animals like the plateau pika upregulate heme-related genes to maintain adequate blood oxygen levels (62). The elevated heme content in high-altitude pikas may be a factor inducing the mutations in the *Hbp* of the isolate in this study. It is important to acknowledge that this study has certain limitations. Although a relatively high number of mutation sites were identified in the *Hbp* gene of *B. grahamii* isolates from the Qinghai-Tibet Plateau, the limited sample size prevents us from determining whether these mutations are specifically caused by the unique environmental conditions of the region.

### Conclusions

This study revealed a classification of *Bartonella* species based on their virulence factor profiles. In the high virulence factor group, besides the well-known *B. henselae* and *B. quintana*, other species such as *B. kosoyi*, *B. koehlerae*, and *B. tribocorum* were also included. Group membership suggests potential common molecular bases for their pathogenicity. However, their actual pathogenicity is also modulated by multifaceted factors, including host factors, transmission routes, and ecological contexts. Some relatively younger *Bartonella* species, during their evolution from the ancient *B. bacilliformis*, gradually acquired *Trw* and *VirB/VirD4*, thereby replacing some of the functions of the original flagella and substituting the *flagella* virulence factor. This suggests that *Bartonella* evolved to adapt to a more intracellular parasitic lifestyle by modifying its virulence factor profile over time. Additionally, this study found that the infection rate of *B. grahamii* in Plateau Pikas from the Menyuan region of the Qinghai-Tibet Plateau was 12%. The isolated strains contain a significant number of unique genes of unknown function, suggesting the uniqueness of the Qinghai-Tibet Plateau strain. The isolated strains contain 14 amino acid mutations in the virulence factor, *hbp*, which may be induced by the unique physiological characteristics of the Plateau Pika on the Qinghai-Tibet Plateau. This highlights the potential role of host-specific evolution in driving unique genomic and virulence factor traits in pathogens.

## MATERIALS AND METHODS

### Sampling

The plateau pikas were captured around the villages of Menyuan County (located at approximately 37.430698 °N, 101.539072 °E) in the northern part of the Qinghai-Tibet Plateau (based on GB/T 2260-2007) (63). Plateau pikas were captured using snare traps, humanely euthanized, and dissected to collect tissue specimens of the heart, liver, spleen, lungs, and kidneys. All tissue samples were immediately stored in cryovials immersed in liquid nitrogen and subsequently transported to the laboratory for analysis. Samples were stored in a freezer at −80°C until the tests began.

### *Bartonella* identification

The tissues were crushed and homogenized with the SCIENTZ-48L Frozen High-Throughput Tissue Grinder (Xinzhi, Ningbo, China). Then the DNA was extracted using a QIAamp DNA Mini Kit (QIAGEN, Hilden, Germany) according to the manufacturer’s protocol. Extracted DNA was used for amplification of *16S-23SrRNA ITS* (455 bp), *gltA* (379 bp), and *rpoB* (852 bp), and the primers and PCR conditions are shown in Table S1. Pure water as a negative control was included. The PCR products were purified by agarose gel electrophoresis for sequencing (Sangon, Shanghai, China). The obtained sequences were then compared to those in the public database using BLASTn. The *gltA* and *rpoB* genes, along with their corresponding homologous gene sequences, were aligned using MAFFT (v7.525) (64). Phylogenetic trees for the *gltA* and *rpoB* genes were then constructed using IQ-TREE (v2.3.6) (65), with Model Finder Plus (MFP) used to select the optimal tree-building model. A bootstrap analysis with 1,000 replicates was performed to assess the reliability of the phylogenetic trees. The phylogenetic trees were visualized using the R package ggtree (v3.10.1).

### *Bartonella* isolation

The positive samples identified through PCR were used for *Bartonella* culture and isolation. An approximately 25 mg lung tissue sample was first homogenized by adding 300 µL of brain heart perfusate. Subsequently, 100 µL of the homogenate was inoculated into the trypticase soy agarose medium (TSA) with 5% defibrinated sheep blood. The inoculated plates were then incubated for 4 weeks at 37°C in a temperature-constant incubator with 5% CO_2_. Petri dishes were examined twice a week to monitor the growth rate of the colonies. When bacterial colonies showed morphological characteristics similar to those of *Bartonella*, they were identified as potential candidates. Candidates were selected from each plate for secondary culture and isolation and then were identified by PCR and sequencing as described above.

### WGS and genome annotation

WGS of the extracted *Bartonella* DNA isolates was performed using the whole-genome shotgun strategy, combining second-generation sequencing (Illumina NovaSeq platform) and third-generation sequencing (Oxford Nanopore ONT platform). For data obtained from the Nanopore platform sequencing, Flye (v2.9.1) and Unicycler (v0.5.0) software were used for assembly (66, 67). The process of data assembly included the removal of adapter contamination and filtering of the data using AdapterRemoval (v2.3.1) (68). The filtered reads were subsequently assembled using SPAdes (v3.15.4) and A5-miseq to generate scaffolds and contigs (69, 70). The assembled data were refined using Pilon (v1.24) software to obtain the genome sequence (71). Proksee (v6.0.3) was used to calculate the GC content at different genomic positions and to generate the genomic circular maps (72). FastANI (v1.33) was employed to calculate the nucleotide identity matrix between different genomes (73).

### Pangenome and virulence factor analysis

To conduct the pangenome analysis of *Bartonella*, all available genomes of 8 *B. grahamii* strains and 50 reference genomes of *Bartonella* were downloaded from the NCBI Genome Database (as of 3 July 2024) using the data sets tool (v15.30.0) provided by NCBI (74). First, open reading frame prediction and gene function annotation were performed on the genomes using Prokka (v1.13) (75). Next, gene family clustering was conducted using OrthoFinder (v2.5.4) (76). This software performs homology searches on all included gene sequences from the genomes, forming different gene clusters and counting the occurrence of each gene cluster in different genomes. Gene clusters present in all genomes were labeled as the core genome, those present in only one genome were labeled as the unique genome, and the remaining gene clusters were labeled as the accessory genome. The pangenome and core genome rarefaction curves were constructed using PanGP (v1.0.1) (77). These rarefaction curves are used to illustrate the openness of the species’ genome. The unique genome of the isolates was annotated for Clusters of Orthologous Gene functional classification using eggNOG-mapper (v2.1.12) (78). All shared homologous single-copy genes from the genomes were concatenated and used to construct a phylogenetic tree. The blastp of Diamond (v0.9.14) (79) was used to compare the amino acid sequences encoded by the genes with the virulence factor database set B (VFDB; 4 July 2024) (80) to identify the virulence factors present in the genomes. Based on the similarity of virulence factor profiles among strains, hierarchical cluster analysis was performed using Ward’s minimum variance method. Using the overall dissimilarity in the number of virulence factors between strains as the metric foundation, strains exhibiting highly similar compositional characteristics were progressively aggregated to form a dendrogram (branching tree structure). Within the cluster dendrogram, strains possessing distinctly similar virulence factor profiles were classified into the same virulence factor group by resolving the major branch points (agglomeration units within the tree structure). The alignment and visualization of virulence factor amino acid sequences were performed using the R package gmsa (v1.8.0). The tertiary structures of the proteins were predicted using SWISS-MODEL (81). PSIPRED was used to classify the amino acid sequences of the proteins. POCASA (v1.1) was employed to predict the protein binding sites (59). All the bioinformatics analyses were conducted on a Linux system (v5.10.0-23-amd64).

## Data Availability

The *gltA* and *rpoB* gene sequences of the isolates in this study have been uploaded to GenBank (accession numbers: PP395757–PP395768). The genomic data of the *Bartonella* plateau isolates in this study have also been uploaded to NCBI, with the accession numbers CP172783–CP172784.

## SUPPLEMENTAL MATERIAL

Supplemental tables spectrum.00465-25-s0001.docx Tables S1 to S6.

ASM does not own the copyrights to Supplemental Material that may be linked to, or accessed through, an article. The authors have granted ASM a non-exclusive, world-wide license to publish the Supplemental Material files. Please contact the corresponding author directly for reuse.
