# Supplementary material for: Pan-genome classification of the virulence spectrum of Bartonella and the variation of heme-binding protein virulence factor in isolates from the Tibetan Plateau
Source: Microbiol Spectr. 2025 Nov 11;13(12):e00465-25. doi: 10.1128/spectrum.00465-25 (PMC12673931; doi:10.1128/spectrum.00465-25)
Supplement: Supplemental tables — Tables S1 to S6. [file spectrum.00465-25-s0001.docx]

**Table S1.** Primers and PCR conditions for identification of *Bartonella*.

| Genes detected | Primers | Sequences (5′-3′ ) | Size of PCR products (bp) | PCR conditions (Temperature; C /Time in second) | | |
| --- | --- | --- | --- | --- | --- | --- |
|  |  |  |  | Denaturation | Annealing | Extension |
| ITS | 425s | CCGGGGAAGGTTTTCCGGTTTATCC | 455 | 95/30 | 60/35 | 72/5 |
|  | 1000as | CTGAGCTACGGCCCCTAAATCAGG |  |  |  |  |
| rpoB | rpoB-F | CGCATTGGCTTACTTCGTATG | 852 | 95/30 | 48/45 | 72/5 |
|  | rpoB-R | GTAGACTGATTAGAACGCTG |  |  |  |  |
| gltA | CS443F | GCTATGTCTGCATTCTATCA | 790 | 95/30 | 48/45 | 72/5 |
|  | CS1210R | GATCYTCAATCATTTCTTTCCA |  |  |  |  |
|  | Bhcs. 781p | CGCATTATGGTCGTATTTGTCC | 379 | 95/30 | 46/30 | 72/5 |
|  | Bhcs. 1137n | GCACGATTTGCATCATCATTTTCC |  |  |  |  |

| **Description** | **Scientific Name** | **Total Score** | **Query Cover** | **E value** | | **Per. ident** | | **Acc. Len** | **Accession** |
| --- | --- | --- | --- | --- | --- | --- | --- | --- | --- |
| Bartonella grahamii subsp. shimonis strain 02 chromosome, complete genome | Bartonella grahamii subsp. shimonis | 1413 | 99% | 0 | 97.69 | | 2186791 | | CP123961.1 |
| Bartonella grahamii subsp. shimonis strain 01 chromosome, complete genome | Bartonella grahamii subsp. shimonis | 1408 | 99% | 0 | 97.57 | | 2259370 | | CP123960.1 |
| Bartonella grahamii strain KR28 RNA polymerase beta subunit (rpoB) gene, partial cds | Bartonella grahamii | 1380 | 99% | 0 | 96.96 | | 884 | | JN647927.1 |
| Bartonella grahamii as4aup, complete genome | Bartonella grahamii as4aup | 1369 | 99% | 0 | 96.72 | | 2341328 | | CP001562.1 |
| Bartonella grahamii strain CL25QHWL RNA polymerase beta subunit (rpoB) gene, partial cds | Bartonella grahamii | 1358 | 96% | 0 | 97.37 | | 825 | | MT815371.1 |
| Bartonella grahamii strain CL05QHWL RNA polymerase beta subunit (rpoB) gene, partial cds | Bartonella grahamii | 1358 | 96% | 0 | 97.37 | | 825 | | MT815368.1 |
| Bartonella grahamii strain CL50QHWL RNA polymerase beta subunit (rpoB) gene, partial cds | Bartonella grahamii | 1358 | 96% | 0 | 97.37 | | 825 | | MT815382.1 |
| Bartonella grahamii strain CL46QHWL RNA polymerase beta subunit (rpoB) gene, partial cds | Bartonella grahamii | 1358 | 96% | 0 | 97.37 | | 825 | | MT815380.1 |
| Bartonella grahamii strain CL33QHWL RNA polymerase beta subunit (rpoB) gene, partial cds | Bartonella grahamii | 1358 | 96% | 0 | 97.37 | | 825 | | MT815376.1 |
| Bartonella grahamii strain 2015-81 RNA polymerase beta subunit (rpoB) gene, partial cds | Bartonella grahamii | 1351 | 97% | 0 | 96.79 | | 810 | | MH547328.1 |

**Table S2.** The BLAST alignment results of the *rpoB* gene.

| **Description** | **Scientific Name** | **Total Score** | **Query Cover** | **E value** | **Per. ident** | **Acc. Len** | **Accession** |
| --- | --- | --- | --- | --- | --- | --- | --- |
| Bartonella grahamii strain OC41QH citrate synthase (gltA) gene, partial cds | Bartonella grahamii | 597 | 99% | 3.00E-166 | 99.39 | 372 | KT445923.1 |
| Bartonella grahamii strain OC03QH citrate synthase (gltA) gene, partial cds | Bartonella grahamii | 597 | 99% | 3.00E-166 | 99.39 | 370 | KT445916.1 |
| Bartonella grahamii strain OC68QH citrate synthase (gltA) gene, partial cds | Bartonella grahamii | 592 | 99% | 1.00E-164 | 99.09 | 384 | KT445926.1 |
| Bartonella grahamii strain OC74QH citrate synthase (gltA) gene, partial cds | Bartonella grahamii | 590 | 96% | 5.00E-164 | 100 | 356 | KT445929.1 |
| Bartonella grahamii strain OC42QH citrate synthase (gltA) gene, partial cds | Bartonella grahamii | 590 | 96% | 5.00E-164 | 100 | 362 | KT445924.1 |
| Bartonella grahamii strain OC73QH citrate synthase (gltA) gene, partial cds | Bartonella grahamii | 590 | 96% | 5.00E-164 | 100 | 360 | KT445928.1 |
| Bartonella grahamii strain BART7 citrate synthase (gltA) gene, partial cds | Bartonella grahamii | 588 | 96% | 2.00E-163 | 100 | 357 | MT438463.1 |
| Bartonella grahamii strain OC01QH citrate synthase (gltA) gene, partial cds | Bartonella grahamii | 588 | 96% | 2.00E-163 | 100 | 356 | KT445915.1 |
| Bartonella grahamii strain OC66QH citrate synthase (gltA) gene, partial cds | Bartonella grahamii | 586 | 96% | 6.00E-163 | 100 | 356 | KT445925.1 |
| Bartonella grahamii strain OC29QH citrate synthase (gltA) gene, partial cds | Bartonella grahamii | 586 | 96% | 6.00E-163 | 100 | 355 | KT445920.1 |

**Table S3.** The BLAST alignment results of the *gltA* gene.

**Table S4.** The general genomic characteristics of the *B.grahamii* isolate.

| **Sequence** | **Seq Length (bp)** | **GC Content (%)** | **CDS num** |
| --- | --- | --- | --- |
| Chromosome | 2,197,397 | 38.03 | 1,912 |
| Plasmid | 31,902 | 36.49 | 39 |

**Table S5.** The amino acid mutations occurring in the heme-binding protein of the Bartonella strain.

| Amino acid position | 28 | 67 | 68 | 74 | 75 | 78 | 113 | 121 | 126 | 128 | 151 | 196 | 198 | 222 |
| --- | --- | --- | --- | --- | --- | --- | --- | --- | --- | --- | --- | --- | --- | --- |
| Original Hbp amino acids | P | S | N | A | P | L | N | N | L | D | A | T | S | L |
| Isolate Hbp amino acids | S | A | R | V | S | Y | S | K | V | E | P | S | L | F |
| Types of amino acids | Sn | Sn | P | S→H | Sn | H→A | P→Sn | P | H | P | Sn | Sn | Sn→H | H→A |

**Table S6.** Changes in the binding sites of the heme-binding protein before and after mutation

| Hbp | Pocket Position | Volume | Volume Depth | Average VD |
| --- | --- | --- | --- | --- |
| Original Hbp | 75 | 410 | 1127 | 2.75 |
|  | 414 | 53 | 135 | 2.55 |
|  | 277 | 41 | 448 | 10.94 |
|  | 126 | 28 | 64 | 2.30 |
|  | 43 | 23 | 58 | 2.52 |
|  | 157 | 23 | 92 | 4.01 |
| Isolate Hbp | 69 | 387 | 1048 | 2.71 |
|  | 411 | 53 | 135 | 2.55 |
|  | 277 | 41 | 448 | 10.93 |
|  | 123 | 28 | 64 | 2.30 |
|  | 156 | 23 | 92 | 4.01 |
